# Receptivity to Bariatric Surgery in Qualified Patients

**DOI:** 10.1155/2016/5372190

**Published:** 2016-07-19

**Authors:** Michael Fung, Sean Wharton, Alison Macpherson, Jennifer L. Kuk

**Affiliations:** ^1^School of Kinesiology, York University, Toronto, ON, Canada M3J 1P3; ^2^The Wharton Medical Clinic, Hamilton, ON, Canada L8L 5G8

## Abstract

*Objectives*. Bariatric surgery has been shown to be an effective intervention for weight loss and diabetes management. Despite this, many patients qualified for bariatric surgery are not interested in undergoing the procedure. The objective of this study is to determine the factors influencing receptivity to bariatric surgery among those who qualify for the procedure.* Methods*. Patients attending a publicly funded weight management clinic who qualified for bariatric surgery were asked to complete an elective questionnaire between February 2013 and April 2014.* Results*. A total of 371 patients (72% female) completed the questionnaire. Only 87 of 371 (23%) participants were interested in bariatric surgery. Individuals interested in bariatric surgery had a higher BMI (48.0 versus 46.2 kg/m^2^, *P* = 0.03) and believed that they would lose more weight with surgery (51 versus 44 kg, *P* = 0.0069). Those who scored highly on past weight loss success and financial concerns were less likely to be interested in bariatric surgery, whereas those who scored highly on high receptivity to surgery and positive social support were more likely to be interested in bariatric surgery.* Conclusion*. Although participants overestimated the effect of bariatric surgery on weight loss, most were still not interested in bariatric surgery.

## 1. Introduction

In Canada, the rate of overweight and obesity in the adult population was 62.1% and 25.4%, respectively [1]. Obesity is demonstrated to be associated with a variety of conditions and comorbidities including stroke, dyslipidemia, type 2 diabetes, hypertension, osteoarthritis, and increased mortality risk [2, 3] as well as higher health care expenditure [4].

As a modest weight loss of 5–10% body weight is demonstrated to improve fasting plasma glucose, HbA1c, cardiovascular disease risk, and other obesity related comorbidities [5], Canadian guidelines recommend weight loss for overweight or obese adults [3]. However, traditional methods of weight loss including diet, physical activity, and pharmacotherapy interventions have been shown to be ineffective when considering follow-up lengths of greater than 1 year [6–8]. Bariatric surgery, comparatively, has been shown to be a more effective intervention for weight loss, diabetes management, dyslipidemia, and reducing mortality risk [9–11]. However, many individuals qualified for bariatric surgery have negative impressions about the efficacy and safety of surgical weight loss interventions [12, 13].

As such, this study aims to identify the factors associated with receptivity to bariatric surgery in qualified individuals. This expands on previous research by examining the motivating factors behind those who are interested as well as those not interested in bariatric surgery.

## 2. Methods

The Wharton Medical Clinic (WMC) is a referral based weight and diabetes management clinic. Bariatric surgery is not performed at WMC; however WMC physicians provide consultations on bariatric surgery for qualified patients and are able to make referrals if required. All participants freely gave their full informed written consent for the use of questionnaire and medical data for research purposes. The study protocols were reviewed and approved by York University's Ethics Review Board.

Participants selected for this study were qualified for bariatric surgery according to NIH recommendations with a BMI of 40 kg/m^2^ or greater or a BMI of 35 kg/m^2^ or greater and obesity related comorbidities. An opportunistic sample of patients who attended the clinic and qualified for bariatric surgery covered under the provincial health care plan was approached to complete the study questionnaire. A total of 400 questionnaires were completed by patients who gave their informed consent to participate in this research. Participants with missing height or weight measurements were excluded from this analysis (*n* = 29).

Differences between bariatric surgery interest groups were assessed using *t*-tests and chi-square test for continuous and categorical variables, respectively. Survey responses regarding receptivity to bariatric surgery based on rating scale (1 = strongly disagree and 5 = strongly agree) were categorized into four groups: past weight loss success, receptivity to surgery, positive social support for surgery, and financial concerns regarding surgery. Where necessary, scores were inverted to match category direction. Once categorized, scores were summated and standardized to a scale of 5. Poisson regression was used to determine the relative risk between interest in bariatric surgery and the four thematic categories. This analysis was adjusted for BMI, age, and sex. Data was analyzed using SAS 9.4 with statistical significance set at *P* < 0.05.

## 3. Results

Table 1 presents the characteristics of the sample (*N* = 371). Only 87 (23%) participants were interested in bariatric surgery. Participants had a mean age of 48.0 ± 11.4 years, weight of 136.4 ± 25.7 kg, and BMI of 48.4 ± 6.8 kg/m^2^. The prevalence of type 2 diabetes was 11%. The sample is predominately white (79%) and female (72%). There were no significant differences in sex, ethnicity, age, or prevalence of type 2 diabetes between those interested and not interested in bariatric surgery (*P* > 0.05). Individuals interested in bariatric surgery had a higher BMI than those not interested (48.0 versus 46.2 kg/m^2^, *P* = 0.03). Additionally, individuals interested in bariatric surgery believed that they would lose more weight with surgery than those not interested (51 versus 44 kg, *P* = 0.0069, or 37% versus 33% of body weight, *P* = 0.01).

The top cited reasons for not being interested in bariatric surgery (Table 2) are fear of other complications from surgery (51.1%), the fact that they did not need surgery to lose weight (32.0%), and fear of dying (24.6%). Among those not interested in bariatric surgery, only 7.7% of respondents stated that they did not believe that bariatric surgery would work. The top cited reasons for being interested in bariatric surgery (Table 2) are health benefits (94.3%), greater weight loss (89.7%), and improved mobility (85.1%).

The mean scores for Likert scale questions regarding bariatric surgery are listed under the 4 categories and presented in Table 3. After adjusting for BMI, sex, and age, those who scored high in past weight loss success (RR = 0.71 (0.57–0.87)) and financial concerns regarding surgery (RR = 0.49 (0.40–0.60)) were less likely to be interested in bariatric surgery while those who scored high in receptivity to surgery (RR = 1.90 (1.56–2.31)) or positive social support (RR = 1.86 (1.51–2.29)) were more likely to be interested in bariatric surgery (Table 3).

## 4. Discussion

To our knowledge this is the first study to demonstrate factors associated with receptivity to bariatric surgery in qualified patients within a publicly funded clinic. Individuals who were interested in bariatric surgery had a higher BMI and greater weight loss expectations for surgery than those not interested. Those who report past weight loss success or financial concerns were less likely to be interested in bariatric surgery while those with high receptivity to surgery or positive social support regarding surgery were more likely to be interested in bariatric surgery.

Bariatric surgery is the most effective weight loss intervention currently available. However, the wait times for this procedure are currently the longest for any surgically treated condition in Canada [14]. Even with the low levels of interest in bariatric surgery reported in our study, there are still wait times of greater than 5 years for bariatric surgery in Canada [14]. To ensure that patients are aware of the interventions available to them, all participants at the clinic underwent personalized physician consultation for bariatric surgery. Those interested in bariatric surgery had a modestly higher BMI than those not interested (48.0 versus 46.2 kg/m^2^). Interestingly, this 4% difference is nearly the amount that would be considered a clinically significant weight loss [3, 5]. It appears that those who were not interested in bariatric surgery are not driven by a lack of belief in effectiveness of surgery as only 7.7% of these individuals stated that they did not believe that bariatric surgery would work but believed that they would lose less weight than those interested in surgery. However, both groups had high weight loss expectations (37% versus 33% of body weight, resp.) that are in line with general desired weight loss expectations in patient populations [15], but higher than the 20–32% weight loss typically observed with bariatric surgery [9, 16]. However, it is important to note that the longer term 16–23% weight loss associated with bariatric surgery would fall in the disappointing range despite being much higher than the 5–10% body weight loss recommended for health benefits [3, 5]. Thus, it appears that it may not be a perceived lack of effectiveness in bariatric surgery as a weight loss intervention that is driving the high level of disinterest in the procedure.

Consistent with previous studies, a large proportion of qualified patients are not interested in surgical interventions for weight management [12]. Interestingly, participants in the not interested group were more likely to agree with the statement “I have lost weight in the past but cannot keep it off.” Although this seems counterintuitive, it is possible that the ability to lose weight, despite repeated weight regain, creates a sense that a surgical intervention is not necessary to achieve what is perceived to be achievable through less intrusive interventions. Alternatively, these individuals may in fact be more successful in past weight loss attempts as indicated by their lower BMI. However, previous studies generally report low levels of successful weight loss maintenance (5–21% success) [17, 18]. This reflects a large disparity between what should be realistically expected from both surgical and nonsurgical weight loss interventions and what patients believe is actually achievable [19]. This may suggest that greater emphasis must be placed on educating patients on realistic weight management expectations.

All participants agreed that surgery would cause both a drastic change in eating habits and lifestyle. However, those who agreed more strongly with statements in this category were more likely to be interested in bariatric surgery. This may suggest that those who are interested in surgery may be more aware of the diet and lifestyle changes necessary following bariatric surgery or those who are not interested may have already drastically altered their eating habits and lifestyle as reflected by their lower BMI, and thus the requirements following bariatric surgery would not be considerably different. Previous studies suggest that these lifestyle changes can be difficult to maintain, and even with bariatric surgery, the majority of patients report noncompliance with at least one behavioural recommendation after surgery [20]. Furthermore, fear of other complications due to surgery, which included lifestyle changes, was the top reason cited by respondents not interested in bariatric surgery. Despite possible complications, bariatric surgery is a relatively safe procedure. In a study of 4776 patients undergoing bariatric surgery, 0.3% of patients died within 30 days of surgery, while 4.1% of patients had a severe adverse outcome [21]. Comparatively, the 30-day mortality risk for hip or knee replacement is higher at approximately 1% [22]. On the other hand, patients who underwent bariatric surgery had a 89% reduction in relative risk of death (0.68% versus 6.17%) over a 5-year follow-up [23]. Although it is realistic to have concerns regarding any surgical procedure, it is possible that greater emphasis should be placed on the relative safety and overall health benefits of this intervention.

Although bariatric surgery has been demonstrated to be a relatively safe and effective method of weight loss and intervention for many obesity related comorbidities [9–11, 21], there is still resistance to surgery in many patients [12]. In a study examining 77 patients with severe obesity attending a clinic for routine outpatient appointments or hospitalization for other reasons, Afonso et al. report that 57% were not interested in bariatric surgery [12], while a study examining patients with severe obesity and obstructive sleep apnea found that 64% were not interested in bariatric surgery [24]. Our study found even more extreme results, with 77% of qualified participants not interested in bariatric surgery. As the participants in the current study are participating in a weight management clinic, it is surprising that there is less receptivity to bariatric surgery in our sample. However, as health benefits were the number one reason respondents cited for being interested in bariatric surgery, it is possible that the higher level of interest is due to the greater prevalence of nonspecific health issues in the Afonso study which recruited participants from patients attending clinics or hospitals for reasons other than obesity [12]. Interestingly, although bariatric surgery has been demonstrated to be an effective treatment for the remission of type 2 diabetes [10, 25] and for improved recovery rates from hypertension [26, 27], there was no difference in prevalence of type 2 diabetes or hypertension between those who were interested and those who were not interested in bariatric surgery. This may suggest that although participants are aware of the weight loss benefits, perhaps they are not fully aware of the value of bariatric surgery as an intervention for diabetes and other obesity related comorbidities.

Hesitance to consider bariatric surgery has been demonstrated in a previous study, with the majority of respondents holding negative impressions of bariatric surgery [28]. To our knowledge, this is the first study demonstrating that positive social support is related with interest in bariatric surgery itself. Previous studies report a nonsignificant trend towards increased weight loss in bariatric surgery patients with positive social support [29, 30]. Thus, strong positive social support may be important for both bariatric surgery interest and success. It is possible that considering bariatric surgery for these participants is internally viewed as a failure at previous interventions and positive social support helps ameliorate these concerns.

A previous study reports that insurance coverage did not appear to be a driving factor for patients interest in bariatric surgery [31]. However, the authors cautioned that these patients were all offered coverage by their insurance for bariatric surgery and thus were perhaps not generalizable to all individuals. Our participants also qualified for bariatric surgery coverage under the Ontario Health Insurance Plan. Nevertheless, cost was one of the top 5 reasons cited as a barrier. It is possible that there are extraneous costs to the actual surgery itself, such as taking time off work, additional childcare costs, or the cost of lifestyle adjustments that are factoring into these individuals receptivity to surgery. This suggests that the interest in bariatric surgery in populations without health care coverage may be even lower than the rates observed here. Currently, rates of interest for bariatric surgery have not been reported in nationally representative samples.

Several strengths and limitations of the present study warrant mention. The majority of the sample was white and female and thus may not be generalizable to the general population. Future studies should examine differences in quality of life and current levels of physical activity. A strength of this study was the larger sample size used in the analysis compared to previous studies (*n* = 371 versus 44 and 77) [12, 15]. All individuals in our sample actively attended a medical clinic that is predominately focused on lifestyle weight management and received bariatric surgery consultations and education. Thus, patients attending this weight management clinic may be more hesitant to undergo surgery than the general eligible population. However, misconceptions regarding bariatric surgery are also likely to be even greater among the general population which generally perceives bariatric surgery as negative and unsafe [28].

Our study suggests that several key issues require greater education regarding weight management goals and surgical interventions. Although those interested in bariatric surgery had a higher BMI than those not interested, both groups still have unrealistic expectations regarding bariatric surgery as a weight loss intervention. It appears that weight loss expectations are not a major driving factor of interest in bariatric surgery, as there are still very low levels of interest in bariatric surgery.

## Figures and Tables

**Table 1:** Respondent characteristics in 371 patients of the Wharton Medical Clinic who qualify for bariatric surgery.

	Interested in bariatric surgery	Not interested in bariatric surgery	*P* value
*N*, %	87 (23)	284 (77)	—
Female, %	67 (77)	200 (70)	0.23
White, %	63 (72)	236 (83)	0.08
Age, years	47.5 (11.2)	48.2 (11.5)	0.61
Type 2 diabetes, %	14 (16)	26 (9)	0.17
BMI, kg/m^2^	48.0 (8.2)	46.2 (6.6)^*∗*^	0.05
Hypercholesterolemia, %	7 (8)	37 (13)	0.14
Hypertension, %	56 (64)	99 (35)	0.9
Obstructive sleep apnea, %	2 (2)	12 (4)	0.34

Data are presented as frequency (%) or mean (SD).

^*∗*^Significantly different from being interested in bariatric surgery (*P* < 0.05).

**Table 2:** Reasons respondents cited for being interested or not interested in bariatric surgery.

Interested in bariatric surgery (*n* = 87)		Not interested in bariatric surgery (*n* = 284)	
Health benefits, %	82 (94.3)	Fear of other complications from surgery, %	145 (51.1)
Greater weight loss, %	78 (89.7)	Do not need surgery to lose weight, %	91 (32.0)
Improve mobility, %	74 (85.1)	Fear of dying, %	70 (24.6)
Aesthetics appearance, %	44 (50.6)	Fear of surgery in general, %	68 (23.9)
Diabetes management, %	26 (29.9)	Cost, %	58 (20.4)
		Pain, %	39 (13.7)
		Do not believe it will work, %	22 (7.7)
		Fear of judgment, %	9 (3.2)
		Religious or cultural reasons, %	2 (0.7)

**Table 3:** Mean scores for responses to questions regarding receptivity to bariatric surgery.

	Interested	Not interested	Relative risk (95% CI)
*Past weight loss success*	*2.3 (0.6)*	*2.5 (0.6)* ^*∗*^	*0.71 (0.57–0.87)*
Dieting has helped me manage my weight in the past	2.7 (1.2)	3.3 (1.2)^*∗*^	0.74 (0.63–0.87)
Exercise has helped me manage my weight in the past	3.1 (1.1)	3.6 (1.2)^*∗*^	0.77 (0.66–0.90)
Weight loss medication has helped me manage my weight in the past	2.0 (1.1)	1.9 (1.2)	1.07 (0.92–1.25)
Diets are easy to stick to	1.9 (0.9)	2.2 (1.0)	0.81 (0.66–1.00)
Exercise is easy to stick to	2.2 (1.0)	2.5 (1.1)	0.83 (0.69–0.99)
I have lost weight in the past but cannot keep it off (i)	1.7 (1.2)	2.2 (1.1)^*∗*^	Not estimable

*Receptivity to surgery*	*2.7 (0.5)*	*2.1 (0.6)* ^*∗*^	*1.90 (1.56–2.31)*
Surgery will help maintain my current weight	2.7 (1.5)	2.3 (1.1)^*∗*^	1.80 (1.63–1.99)
Surgery would be a last resort (i)	2.8 (1.3)	1.5 (1.1)^*∗*^	1.79 (1.64–1.96)
Surgery will cause a drastic change in my eating habits (i)	1.8 (1.0)	2.2 (1.3)^*∗*^	0.79 (0.65–0.96)
Surgery will cause a drastic change in my lifestyle (i)	1.8 (1.1)	2.3 (1.3)^*∗*^	0.74 (0.61–0.90)
I believe surgery will be effective in helping me reach my goal weight	4.5 (0.8)	2.7 (1.3)^*∗*^	2.85 (2.21–3.68)

*Positive social support regarding surgery*	*3.5 (1.2)*	*2.7 (0.9)* ^*∗*^	*1.86 (1.51–2.29)*
My family approves of surgery	3.8 (1.3)	2.5 (1.2)^*∗*^	1.75 (1.48–2.08)
My partner approves of surgery	3.9 (1.3)	2.4 (1.2)^*∗*^	1.85 (1.53–2.22)
My friends approve of surgery	3.7 (1.2)	2.6 (1.1)^*∗*^	1.76 (1.49–2.07)
People will judge me if I get bariatric surgery (i)	2.3 (1.2)	2.3 (1.3)	1.02 (0.87–1.19)

*Financial concerns regarding surgery*	*1.8 (0.7)*	*2.6 (0.8)* ^*∗*^	*0.49 (0.40–0.60)*
I would get surgery if the costs were covered (i)	1.5 (1.0)	3.6 (1.4)^*∗*^	0.40 (0.31–0.52)
I would pay for surgery myself	2.2 (1.2)	1.6 (1.0)^*∗*^	1.36 (1.20–1.56)

Data are presented as mean (SD) and as relative risk (95% confidence interval) adjusted for BMI, sex, and age. Scores are on a scale of 1 (strongly disagree) to 5 (strongly agree). (i) signifies inverted score for category score calculation (i.e., 1 = strongly agree and 5 = strongly disagree).

^*∗*^Significantly different from being interested in bariatric surgery (*P* < 0.05).
